# Simple Analytical Approximations for Donnan Ion Partitioning in Permeable Ion-Exchange Membranes Under Reverse Electrodialysis Conditions

**DOI:** 10.3390/membranes15120365

**Published:** 2025-12-01

**Authors:** Antonio Ángel Moya

**Affiliations:** Departamento de Física, Universidad de Jaén, 23071 Jaén, Spain; aamoya@ujaen.es

**Keywords:** reverse electrodialysis, ionic partitioning, Donnan electric potential, renewable energy, ion-exchange membranes

## Abstract

Reverse electrodialysis (RED) is a relatively recent technology for renewable energy harvesting from the interaction of river and seawater. This paper revisits the thermodynamic equilibrium governing the ionic transport processes through ion-exchange membranes (IEMs) under RED conditions and theoretically derives approximate analytical expressions for the ionic concentrations at the inner boundaries of a permeable membrane with well-stirred baths. The equation for the Donnan ion partitioning at the membrane–solution interface, which is based on the equality of the electrochemical potential in the two phases, is analysed for binary salts with symmetric (1:1) and asymmetric (2:1) electrolytes, by considering bathing solutions with the equivalent concentrations 0.02 M in the dilute bath, and 0.5, 1, and 1.5 M in the concentrate one. Simple approximate analytical expressions exhibiting the evolution with the membrane fixed-charge concentration of the counter-ionic concentrations at the inner boundaries of the membrane, the concentration gradients inside the membrane, the total Donnan electric potential, and the ionic partitioning coefficients have been derived. The approximate generalised expressions for a general *z*_1_:*z*_2_ binary electrolyte are also presented for the first time.

## 1. Introduction

Electrodialysis (ED) and reverse electrodialysis (RED) are electro-membrane processes that play central roles in desalination, wastewater treatment, and energy harvesting from salinity gradients. The increasing global demand for freshwater and renewable energy continues to drive innovation and accelerate advancements in those technologies [1]. While ED is a well-established technique, current research is increasingly focused on boosting its sustainability and aligning with circular economy principles through membrane recycling and system miniaturization. RED, though still emerging, offers exciting potential for low-carbon energy generation [2], particularly when coupled with hybrid technologies such as Reverse Osmosis (RO) [3].

RED is a relatively recent technology for harvesting renewable energy from the interaction of river and seawater [4,5,6,7]. The salinity difference between these two water sources enables the selective transport of counter-ions from seawater to freshwater through an ion-exchange membrane (IEM), creating a voltage drop across the membrane that can power an external electrical device [8,9,10,11,12,13,14,15,16]. Current research is increasingly focused on understanding ionic transport through IEMs separating electrolyte solutions of differing salinities due to the rapid developments and promising future applications of this technology.

The Nernst–Planck (NP) analysis is the most widely used theoretical treatment to describe the transport processes of ionic species though IEMs on the basis of the Teorell–Meyer–Sievers model [17]. This analysis is also used when describing the ionic transport processes in a RED stack including hydrodynamic conditions in the diffusion boundary layers adjacent to the membranes [18,19,20,21]. The ionic concentration gradients in the IEMs play a central role in describing the behaviour of a permeable membrane under salinity gradients in RED stacks. They allow us to obtain the open circuit voltage (OCV) and the membrane resistance, which are key parameters to quantify the electric power density and to evaluate the performance of a RED stack [22]. The usual assumption in highly charged membranes by which the co-ion concentration is zero and that of the counter-ion is the equivalent concentration of the fixed-charge of the membrane [23], which is known as the total co-ion exclusion assumption [24], is not meaningful in permeable membranes under RED conditions. This is because the ionic concentrations in the bathing solutions take different values corresponding to the concentrate and dilute baths, and then the assumption would deal to zero ionic concentration gradients in the IEM, which only allows us to consider ohmic potential differences across the membrane. The Donnan equilibrium equation for the ionic partitioning at each membrane–solution interface must be analytically or numerically solved to obtain the ionic concentrations at the inner boundaries of the membrane [25]. This is particularly interesting in multi-ionic systems to assess the power loss due to the uphill transport of divalent ions [26,27]. Recently, interesting studies have used advanced models to include activity coefficients and steric effects in those surveys [28,29,30,31,32,33]. However, although there is real analytical solution for the ionic concentrations in IEMs with symmetric binary electrolytes, simple approximate analytical expressions, particularly those applicable to the concentrate bath, have been not explored enough.

The objective of this paper is to revisit the thermodynamic equilibrium governing the ionic transport processes through permeable IEMs with well-stirred baths under RED conditions and theoretically derive approximate analytical expressions for the ionic concentrations at the inner boundaries of the membrane, which are subsequently compared with exact solutions to evaluate their accuracy and applicability. The equation for the Donnan ion partitioning at the membrane–solution interface, which is based on the equality of the electrochemical potential in the two phases, is analysed for binary electrolytes of both symmetric (1:1) and asymmetric (2:1) types, by considering bathing solutions with the equivalent concentrations 0.02 M in the dilute bath and 0.5, 1, and 1.5 M in the concentrate one. Firstly, the ion partitioning equations expressed by using the counter-ionic concentration as the variable are expanded, and simple approximate analytical expressions for the counter-ion concentration gradients corresponding to small deviations from ideal behaviour are derived. Then, approximate expressions for the ionic concentrations at the inner boundaries of the membrane are obtained. The relative error in counter-ion concentrations when replacing the exact solution for a 1:1 electrolyte with the approximate solution and that of the total co-ion exclusion hypothesis is evaluated as a function of the membrane fixed-charge concentration. Secondly, the total Donnan electric potential, which results in the partial exclusion of co-ions providing the basis of the membrane permselectivity, is analysed at each membrane–solution interface. Finally, simple approximate expressions exhibiting the evolution with the membrane fixed-charge concentration of the ionic partitioning coefficients are derived. The approximate generalised expressions for a general *z*_1_:*z*_2_ binary electrolyte are also presented for the first time.

## 2. Ionic Partitioning in Permeable IEMs

Let us consider a membrane placed between two well-stirred bathing solutions constituted by a mixture of the binary salts with *N* ionic species of valence *z_i_* (*i* = 1, …, *N*). The membrane is an IEM of cationic type, i.e., a membrane having a fixed charge with negative sign. We will denote by *c_i_*_0*K*_ the molar concentrations (mol/m^3^) of ion *i* in the bathing solution *K* (*K* = *L* for the left and *K* = *R* for the right), which obeys the electroneutrality condition of each solution:

(1) $$\sum_{i\;=\;1}^{N}z_{i}\;c_{i0K}\;=\;0$$

At the equilibrium state of the system, the electrochemical potential of each ionic species in both the membrane and solution phases is assumed to be equal. Consequently, the ionic partitioning at the interface *K* can be modelled by applying the Donnan equilibrium conditions given by the extended equation:

(2) $$c_{iK}\;=\;c_{i0K}\;\left(\frac{\gamma_{i}^{S}}{\gamma_{i}^{M}}\right)\;\Phi_{i}\;\exp\;\left(\;-\;\frac{z_{i}\;F}{R\;T}\;\phi_{DK}\right)$$

where *c_iK_* is the molar concentration (mol/m^3^) of ion *i* at the inner boundary of the membrane of the interface *K*; $\gamma_{i}^{S}$ and $\gamma_{i}^{M}$, respectively, are the activity coefficients of ion *i* in the solution and membrane phases; *Φ_i_* is the non-electrostatic partitioning coefficient of ion *i* accounting for the steric effects in the systems; *ϕ_DK_* is the Donnan electric potential (V) at the interface *K*; *F* is the Faraday constant (C/mol); *R* is the gas constant (J/mol·K); and *T* is the absolute temperature (K). It should be noted that this analysis ignores the phenomenon of concentration polarization in the diffusion boundary layers adjacent to the membrane when considering well-stirred baths to equate the interfacial concentrations to the bulk values.

If *X* stands for the fixed-charge concentration inside the membrane (mol/m^3^), the electrical neutrality condition at the interface *K* can be written as follows:

(3) $$\sum_{i\;=\;1}^{N}z_{i}\;c_{iK}\;=\;X$$

It must be noted that *X* is usually expressed as a function of experimental data as follows:

(4) $$X\;=\;\omega \;\chi \;=\;\omega \;\frac{IEC}{SD}\;\rho$$

where *ω* is the valence of the fixed-charge groups, *χ* is the concentration of fixed-charge groups (mol/m^3^), SD is the swelling degree (kg water/kg dry membrane), *ρ* is the water density (kg/m^3^), and IEC is the ionic exchange capacity (mol·eq/kg). Here, we will consider homogeneous distributions of the fixed charge in the membrane, although inhomogeneous distributions with fixed-charge concentrations depending on position [34] and distributions due to ionic adsorption processes [35] are often found in the literature.

Therefore, if the partitioning coefficient at the interface *K*, *k_K_*, is defined as follows:

(5) $$k_{K}\;=\;\exp\;\left(\;-\;\frac{F}{R\;T}\;\phi_{DK}\right)$$

the extended equation for the ion partitioning is

(6) $$\sum_{i\;=\;1}^{N}z_{i}\;c_{i0K}\;\left(\frac{\gamma_{i}^{S}}{\gamma_{i}^{M}}\right)\;\Phi_{i}\;k_{K}^{z_{i}}\;=\;X$$

which should consider models to describe non-ideal behaviours in membranes [36]. In this work, we are only interested in deriving analytical approximations for ionic concentrations based on the relationship between those in the bathing solutions and the fixed-charge concentration of the membrane [37]. Therefore, we will consider the activity coefficients, as well the coefficient for steric effects, to be unity ($\gamma_{i}^{S}$ = 1, $\gamma_{i}^{M}$ = 1, *Φ_i_* = 1).

Now, the total Donnan electric potential, *ϕ_D_*, is obtained from the Donnan potentials at each interface, *ϕ_DK_*. Then, by evaluating it from the equilibrium of the ion with the valence *z_i_*, it can be written as follows:

(7) $$\phi_{D}\;=\;\phi_{DL}\;-\;\phi_{DR}\;=\;\frac{R\;T}{z_{i}\;F}\;\left[\ln\;\left(\frac{c_{iL}}{c_{i0L}}\right)\;+\;\ln\;\left(\frac{c_{i0R}}{c_{iR}}\right)\right]\;=\;\frac{R\;T}{z_{i}\;F}\;\left[\ln\;\left(\frac{c_{iL}}{c_{iR}}\right)\;+\;\ln\;\left(\frac{c_{i0R}}{c_{i0L}}\right)\right]$$

It we account for the Nernst potential, *ϕ_N_*, given by

(8) $$\phi_{N}\;=\;\frac{R\;T}{z_{i}\;F}\;\ln\;\left(\frac{c_{i0R}}{c_{i0L}}\right)$$

the difference between the total Donnan electric potential, *ϕ_D_*, and the Nernst potential, *ϕ_N_*, which represents the maximum potential achievable across the membrane, is as follows:

(9) $$\Delta \phi \;=\;\phi_{D}\;-\;\phi_{N}\;=\;\frac{R\;T}{z_{i}\;F}\;\ln\;\left(\frac{c_{iL}}{c_{iR}}\right)$$

It must be noted that this variation in the Donnan potential is zero when considering the total co-ion exclusion hypothesis, i.e., ideally selective membranes. Also, it must be considered that the OCV of an IEM system in a RED stack is mainly determined by the total Donnan electric potential with the corresponding sign reversal for *c_i_*_0*L*_ > *c_i_*_0*R*_ [22]. Additionally, according to the Teorell–Meyer–Sievers model, the electric potential arising from the contribution of the electric field inside the membrane must be included and added to the Donnan potential to obtain the system potential, even when simplifications are considered [38]. The total electric voltage and power in operando RED stacks are then derived from the OCV and the stack resistance [39].

## 3. Results and Discussion

### 3.1. Symmetric Electrolyte

For a 1:1 binary electrolyte (*N* = 2), such as NaCl, with *z*_1_ = 1, *z*_2_ = −1, and the ionic concentrations in the bathing solutions given by the following equation:

(10) $$c_{10K}\;=\;c_{20K}\;=\;c_{0K}$$

the ionic partitioning coefficient, by considering Equation (6), must be obtained from the numerical solution of the following algebraic equation:

(11) $$k_{K}^{2}\;-\;k_{K}\;\frac{X}{c_{0K}}\;-\;1\;=\;0$$

The solution of this equation gives the well-known ionic partitioning coefficient for a binary electrolyte [21]:

(12) $$k_{K}\;=\;\frac{X}{2\;c_{0K}}\;+\;\sqrt{1\;+\;{\left(\frac{X}{2\;c_{0K}}\right)}^{2}}$$

At the limit *X* → 0 for weakly charged membranes, *k_K_* → 1, such as when corresponding to a neutral membrane. At the limit for strongly charged membranes when *X* >> *c*_0*K*_, this expression leads to

(13) $$k_{K}\;=\;\frac{X}{c_{0K}}$$

which is the solution of the ion partitioning equation under the total co-ion exclusion hypothesis. Now, the counter-ionic concentrations at the inner boundaries of the membrane are

(14) $$c_{1K}\;=\;k_{K}\;c_{10K}\;=\;k_{K}\;c_{0K}$$

and they can be expressed as follows:

(15) $$c_{1K}\;=\;X\;\left[\frac{1}{2}\;+\;\frac{1}{2}\;\sqrt{1\;+\;{\left(2\;\frac{c_{0K}}{X}\right)}^{2}}\right]$$

This result can also be obtained from the solution of the well-known equation:

(16) $$c_{1K}\;c_{2K}\;=\;c_{1K}\left(c_{1K}\;-\;X\right)\;=\;c_{0K}^{2}$$

Equation (15) can now be developed into a Taylor series around the variable *c*_0*K*_/*X*, when it tends to zero, in the following way:

(17) $$c_{1K}\;=\;X\;\left[1+\;{\left(\frac{c_{0K}}{X}\right)}^{2}\;-\;{\left(\frac{c_{0K}}{X}\right)}^{4}\;+\;\ldots \right]$$

**Figure 1:**
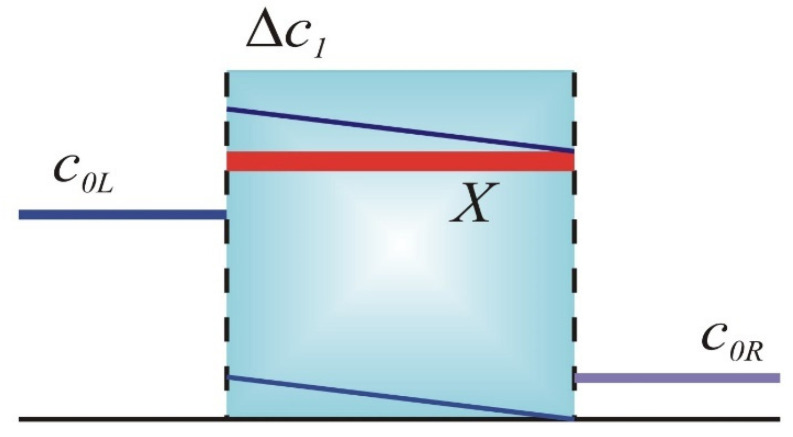
Sketch of an IEM system under RED conditions.

Thus, by retaining terms up to the second order, it follows that

(18) $$c_{1K}\;=\;X\;+\;\frac{c_{0K}^{2}}{X}$$

and the concentration gradient in the IEM (see sketch in Figure 1), which is defined as follows:

(19) $$\Delta c_{1}\;=\;c_{1L}\;-\;c_{1R}$$

can be written as follows:

(20) $$\Delta c_{1}\;=\;\left(k_{L}\;-\;k_{R}\right)\;c_{0k}\;=\;\frac{c_{0L}^{2}}{X}\;-\;\frac{c_{0R}^{2}}{X}$$

This term is positive whatever the value of *X* because *c*_0*L*_ >> *c*_0*R*_, and it tends to zero for the highest values of the membrane fixed-charge concentration, *X*. Then, the counter-ion concentration in the dilute interface can be approximated by *c*_1*R*_ = *X*, while the concentration gradient is as follows:

(21) $$\Delta \;c_{1}\;=\;\frac{c_{0L}^{2}}{X}$$

**Figure 2:**
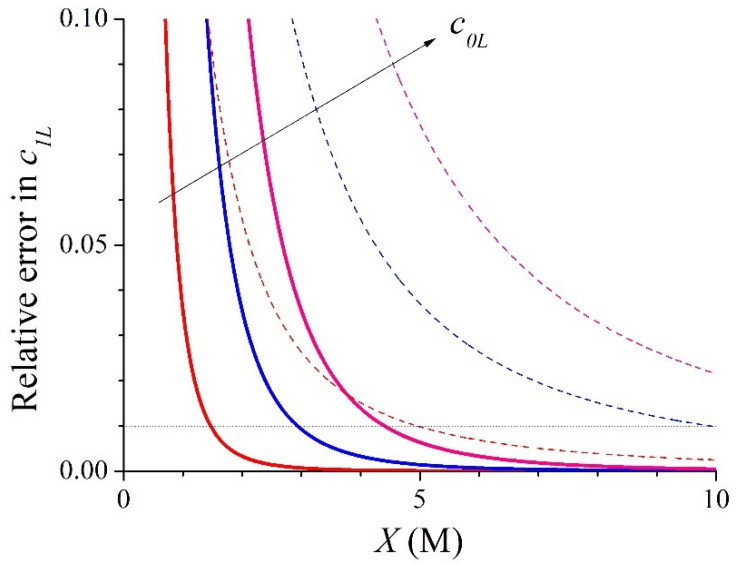
Evolution with *X* of the relative errors in the counter-ion concentration (solid lines) and those obtained when using the total co-ion exclusion hypothesis (dashed lines) for the 1:1 binary system with *c*_0*R*_ = 0.02 M and *c*_0*L*_ = 0.5, 1, and 1.5 M.

The relative error for the counter-ion concentration when considering Equation (18) instead of Equation (15) at the dilute interface with *c*_0*R*_ = 20 mM is very small (<0.16% for *X* > 0.5 M). However, this relative error increases for the concentrate interface. Figure 2 shows the relative error for the counter-ion concentration when taking Equation (18) instead of the exact Equation (15). Now, it is lower than 1% for *c*_0*L*_ = 0.5 M when *X* > 1.45 M, for *c*_0*L*_ = 1 M when *X* > 2.9 M, and for *c*_0*L*_ = 1.5 M when *X* > 4.4 M. In this figure, the relative errors when considering the total co-ion exclusion hypothesis, i.e., the value *X* for the counter-ion concentration instead of Equation (15), have been also plotted by means of dashed lines. Figure 2 shows the significant improvement in the accuracy of the counter-ionic concentration introduced by the approximate expression in Equation (18) with respect to an ideally selective membrane behaviour.

**Figure 3:**
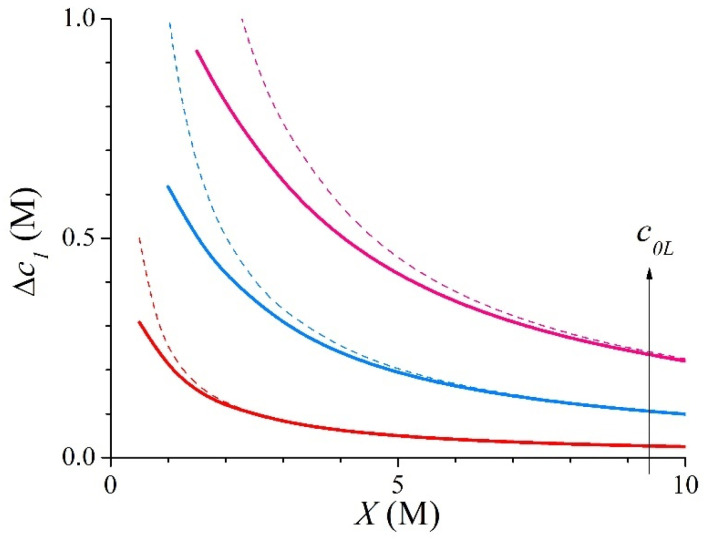
Evolution with *X* of the exact (solid lines) and approximate (dashed lines) values of the ionic concentration gradient for the 1:1 system with *c*_0*R*_ = 0.02 M and *c*_0*L*_ = 0.5, 1, and 1.5 M.

Figure 3 shows the evolution of the counter-ionic concentration gradient inside the membrane with *X* varying from 0.5 M to 10 M for systems with *c*_0*R*_ = 0.02 M and *c*_0*L*_ = 0.5, 1, and 1.5 M. In this figure, the results obtained from the approximate expression given by Equation (21) have also been plotted by means of dashed lines. This figure shows that the cationic concentration gradient increases as the salt concentration in the concentrate bath, *c*_0*L*_, increases. Also, Figure 3 shows that the cationic concentration gradient decreases as the membrane fixed-charge concentration, *X*, increases. The results shown in Figure 3 exhibit an excellent agreement with the approximate expression in Equation (21). The approximate values are higher than the exact ones for each value of *X*. This agreement is better for the highest values of *X* and the smallest values of *c*_0*L*_.

On the other hand, it must be noted that the corresponding approximation for the ionic partitioning coefficient is as follows:

(22) $$k_{K}\;=\;\frac{c_{1K}}{c_{0K}}\;=\;\frac{X}{c_{0K}}\;+\;\frac{c_{0K}}{X}$$

Now, the parameters needed to evaluate the performance of an RED stack require knowing of the diffusion coefficients inside the membrane and the membrane length. Then, for the sake of generality, we will evaluate only the difference between the total Donnan electric potential and the Nernst potential, ∆*ϕ*. This can be approximated by the following equation:

(23) $$\Delta \phi \;=\;\frac{R\;T}{F}\;\left[\ln\;\left(1\;+\;\frac{c_{0L}^{2}}{X^{2}}\right)\right]$$

which, after the series expansion of the logarithmic function, gives the following:

(24) $$\Delta \phi \;=\;\frac{R\;T}{F}\;{\left(\frac{c_{0L}}{X}\right)}^{2}$$

**Figure 4:**
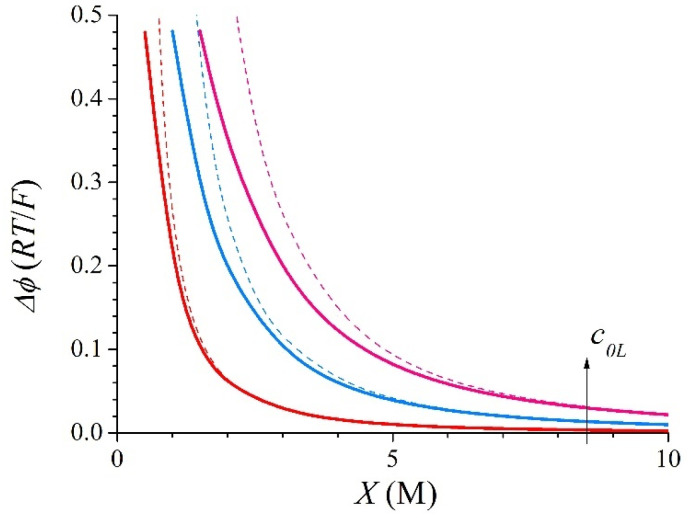
Evolution with *X* of the exact (solid lines) and approximate (dashed lines) values of ∆*ϕ* for a 1:1 binary system with *c*_0*R*_ = 0.02 M and *c*_0*L*_ = 0.5, 1, and 1.5 M.

The Nernst potential for the 1:1 binary electrolyte is given by the following equation:

(25) $$\phi_{N}\;=\;\frac{R\;T}{F}\;\ln\;\left(\frac{c_{0R}}{c_{0L}}\right)$$

and it is negative because *c*_0*L*_ >> *c*_0*R*_ = 0.02 M. In particular, *Fϕ_N_*/*RT* = −3.219 for *c*_0*L*_ = 0.5 M, −3.912 for *c*_0*L*_ = 1 M, and −4.317 for *c*_0*L*_ = 1.5 M. Then, the absolute value of the total Donnan electric potential is lower than the absolute value of the Nernst potential for permeable membranes. Thus, the OCV in an RED stack decreases when employing permeable membranes with values of the fixed-charge concentrations comparable to the concentration of the concentrate bath [22]. Figure 4 shows the evolution with the membrane fixed-charge concentration, *X*, of the difference between the total Donnan electric potential and the Nernst potential, ∆*ϕ*. The terms corresponding to the approximate expressions given by Equation (24) have also been plotted by means of dashed lines. In Figure 4 the excellent agreement between the exact analytical results and those derived from the approximate expressions for the highest values of *X* and the smallest values of *c*_0*L*_ can be clearly observed. It must be observed that, for a given value of *X*, the approximate values of the electric potentials are higher than the exact ones. Also, it must be noted that Equation (24) includes the approximation of the logarithmic function in Equation (23) and it leads to significant relative errors in the electric potential for the lowest values of the membrane fixed-charge concentration.

Finally, Table 1 shows the different approximate expressions and it compares different exact values with the approximate ones, including relative errors, corresponding to the cationic concentrations, the variation of the total Donnan electric potential, and the ionic partitioning coefficient, for *c*_0*L*_ = 0.5 M, which is the usual value for RED with river and sea water. From the obtained results and those for the relative errors shown in Figure 2, we have found that the approximations we have considered can then be used to interpret experimental results in typical IEM systems [40], including RED stacks [41,42,43], because the commercial IEMs generally fall in the range of approximately 1–5 M.

### 3.2. Asymmetric Electrolyte

Results corresponding to the generalization of the above section for *z*_1_:*z*_2_ binary electrolytes can be found in Appendix A. Now, for a 2:1 binary salt, such as MgCl_2_, with *N* = 2, *z*_1_ = 2, *z*_2_ = −1, and the ionic concentrations in the bathing solutions given by the following equations:

(26) $$c_{10K}\;=\;\frac{c_{0K}}{2}$$

(27) $$c_{20K}\;=\;c_{0K}$$

where *c*_0*K*_ represents equivalent ionic concentrations. Then, the ionic partitioning coefficient, by considering Equation (6), must be obtained from the numerical solution of the following third-degree algebraic equation:

(28) $$k_{K}^{3}\;-\;k_{K}\;\frac{X}{c_{0K}}\;-\;1\;=\;0$$

At the limit *X* → 0 for weakly charged membranes, *k_K_* → 1, such as when corresponding to a neutral membrane. At the limit when *X* >> *c*_0*K*_, this expression leads to the following equation:

(29) $$k_{K}\;=\;\sqrt{\frac{X}{c_{0K}}}$$

which is the solution of the ion partitioning equation under the hypothesis of total co-ion exclusion:

(30) $$k_{K}^{2}\;-\;\frac{X}{c_{0K}}\;=\;0$$

Now, the ionic concentrations at the inner boundaries of the membrane are as follows:

(31) $$c_{1K}\;=\;k_{K}^{2}\;c_{10K}\;=\;\frac{1}{2}\;k_{K}^{2}\;c_{0K}$$

Then, for *X* >> *c*_0*K*_, one obtains the well-known result *X*/2 for the cationic concentrations, which can be appropriately used in the inner boundary at the dilute interface.

In order to obtain approximate expressions for the counter-ionic concentration gradient, we will combine Equation (6) and Equation (31) instead of dealing with the analytical solution in a complex variable of Equation (28). Thus, one obtains the following:

(32) $$c_{1K}^{3/2}\;-\;\frac{X}{2}\;c_{1K}^{1/2}=\;{\left(\frac{c_{0K}}{2}\right)}^{3/2}$$

Now, we will write the counter-ionic concentration as follows:

(33) $$c_{1K}\;=\;\frac{X}{2}\;+\;y_{K}$$

and we will use the following series expansions [40]:

(34) $$c_{1K}^{1/2}=\;{\left(\frac{X}{2}\;+\;y_{K}\right)}^{1/2}=\;{\left(\frac{X}{2}\right)}^{1/2}\;+\;\frac{1}{2}\;{\left(\frac{2}{X}\right)}^{1/2}\;y_{K}$$

(35) $$c_{1K}^{3/2}=\;{\left(\frac{X}{2}\;+\;y_{K}\right)}^{3/2}=\;{\left(\frac{X}{2}\right)}^{3/2}\;+\;\frac{3}{2}\;{\left(\frac{X}{2}\right)}^{1/2}\;y_{K}$$

At the limit when *X* >> *c*_0*K*_, i.e., for small deviations of the counter-ion concentration with respect to the ideally selective behaviour, this expression leads to the following:

(36) $$y_{K}\;=\;\sqrt{\frac{c_{0K}^{3}}{4\;X}}$$

Then, the counter-ion concentration gradient in the IEM can be written as follows:

(37) $$\Delta c_{1}\;=\;c_{1L}\;-\;c_{1R}\;=\;\sqrt{\frac{c_{0L}^{3}}{4\;X}}\;-\;\sqrt{\frac{c_{0R}^{3}}{4\;X}}$$

This term is positive whatever the value of *X* because *c*_0*L*_ >> *c*_0*R*_, and it tends to zero for the highest values of the membrane fixed-charge concentration. Then, the counter-ionic concentration in the dilute interface can be approximated by *c*_1*R*_ = *X*/2, while the concentration gradient is as follows:

(38) $$\Delta \;c_{1}\;=\;\sqrt{\frac{c_{0L}^{3}}{4\;X}}$$

**Figure 5:**
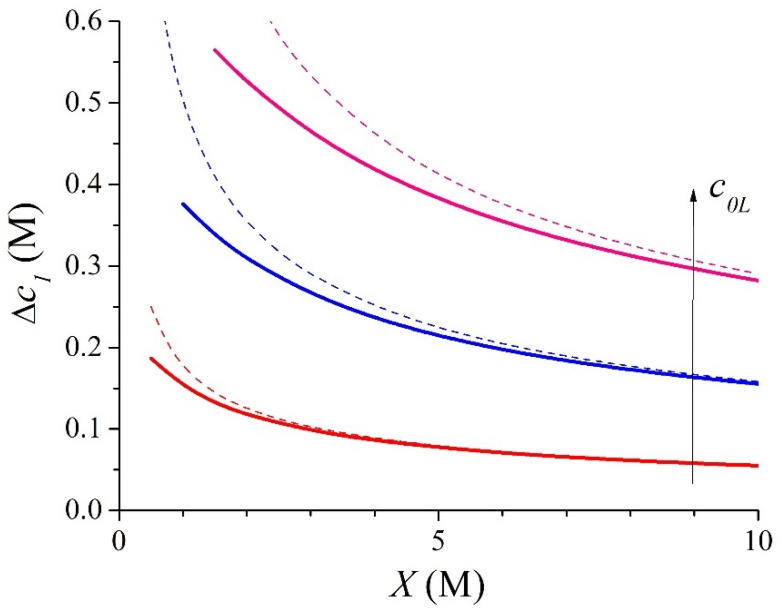
Evolution with *X* of the exact (solid lines) and approximate (dashed lines) values of the ionic concentration gradient for the 2:1 binary system, with *c*_0*R*_ = 0.02 M and *c*_0*L*_ = 0.5, 1, and 1.5 M.

Figure 5 shows the evolution of the counter-ion concentration gradient inside the membrane with the membrane fixed-charge concentration, *X*, varying from 0.5 M to 10 M. The chosen bathing concentrations are *c*_0*R*_ = 0.02 M and *c*_0*L*_ = 0.5, 1, and 1.5 M. The exact values have been obtained by the numerical solution of Equation (32) in a symbolic computation software such as Mathematica (version 13.1), which uses a combination of the Newton–Raphson method and safeguarded line-search strategies to ensure convergence [44]. The approximate expressions given by Equation (38) have also been plotted in this figure by means of dashed lines. Figure 5 shows that the cationic concentration gradient, ∆*c*_1_, increases as the salt concentration in the concentrate bath, *c_0L_*, increases. Also, Figure 5 shows that the concentration gradient decreases as the membrane fixed-charge concentration, *X*, increases. The results shown in Figure 5 exhibit an excellent agreement with the approximate analytical expression in Equation (38). This agreement is better for the highest values of *X* and the smallest values of *c*_0*L*_. Again, the approximate values are higher than the exact ones for each value of *X*.

In this case, the ion partitioning coefficient can be approximated by the following equation:

(39) $$k_{K}\;=\;\sqrt{\frac{X}{c_{0K}}\;+\;\sqrt{\frac{c_{0K}}{X}}}$$

Finally, the difference between the total Donnan electric potential and the Nernst potential can be approximated as follows:

(40) $$\Delta \phi \;=\;\frac{R\;T}{2\;F}\;\left[\ln\;\left(1\;+\;\sqrt{\frac{c_{0L}^{3}}{X^{3}}}\right)\right]$$

which, after the series expansion of the logarithmic function for (*X*/*c*_0*L*_) → 0, gives the following equation:

(41) $$\Delta \phi \;=\;\frac{R\;T}{2\;F}\;{\left(\frac{c_{0L}}{X}\right)}^{3/2}$$

Now, for a binary salt with a divalent cation, the Nernst potential is as follows:

(42) $$\phi_{N}\;=\;\frac{R\;T}{2\;F}\;\ln\;\left(\frac{c_{0R}}{c_{0L}}\right)$$

**Figure 6:**
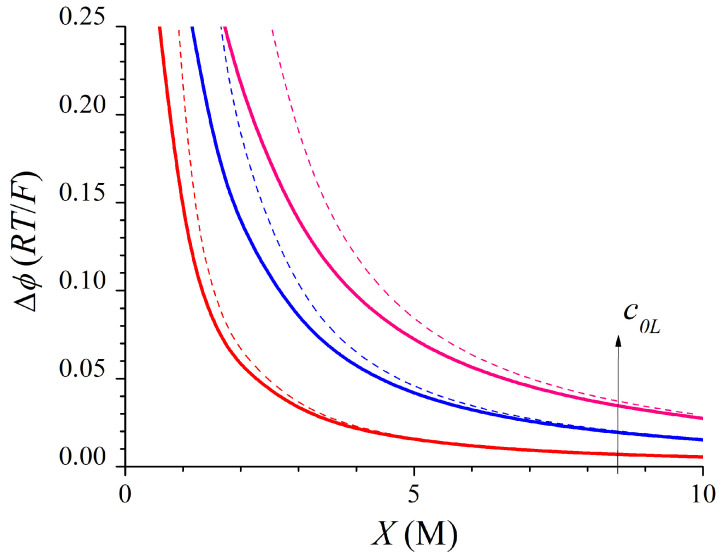
Evolution with *X* of the exact (solid lines) and approximate (dashed lines) values of ∆*ϕ* for the 2:1 binary system with *c*_0*L*_ = 0.5, 1, and 1.5 M and *c*_0*R*_ = 0.02 M.

Again, the Nernst potential is negative because *c*_0*L*_ >> *c*_0*R*_ = 0.02 M. For a divalent counter-ion, one obtains *Fϕ_N_*/*RT* = −1.609 for *c*_0*L*_ = 0.5 M, −1.956 for *c*_0*L*_ = 1 M, and −2.159 for *c*_0*L*_ = 1.5 M. Then, the absolute value of the total Donnan electric potential is lower than that of the Nernst potential for permeable membranes. Thus, the OCV in an RED stack decreases when employing non-ideally selective membranes with values of the fixed-charge concentrations comparable to the ionic concentration of the concentrate bath [22]. Figure 6 shows the evolution with the membrane fixed-charge concentration, *X*, varying from 0.5 M to 10 M, of the difference between the total Donnan electric potential and the Nernst potential. The terms corresponding to the approximate expressions given by Equation (41) have also been plotted by means of dashed lines. In Figure 6 the excellent agreement between the exact results and those derived from the approximate expressions for the highest values of *X* and the smallest values of *c*_0*L*_ can be clearly observed.

Now, Table 2 shows the approximate expressions corresponding to the parameters describing the ion partitioning for the 2:1 asymmetric electrolyte. Also, Table 2 compares different exact values with the approximate ones corresponding to the counter-ionic concentrations, the variation of the total Donnan electric potential, and the ionic partitioning coefficient for *c*_0*L*_ = 0.5 M. From the obtained results, we have found that the relative error in the counter-ionic concentration is lower that 1% for *X* > 1.5 M.

Finally, it is worth highlighting the significant role of the asymmetry of the electrolyte in the ion partitioning equations, such as can be inferred from the results shown in Appendix A. In particular, for a 2:1 electrolyte, such as Na_2_SO_4_ (*N* = 2, *z*_1_ = 1, *z*_2_ = −2, *c*_10*K*_ = *c*_0*K*_, and *c*_20*K*_ = *c*_0*K*_/2) the approximate ion partitioning coefficient is given by the following equation:

(43) $$k_{K}\;=\;\frac{X}{c_{0K}}\;+\;{\left(\frac{c_{0K}}{X}\right)}^{2}$$

which significantly differs from that in Equation (39) for the 2:1 electrolyte. The counter-ionic concentration in the dilute interface can be approximated by *c*_1*R*_ = *X* while the concentration gradient is as follows:

(44) $$\Delta \;c_{1}\;=\;X\;{\left(\frac{c_{OL}}{X}\right)}^{3}$$

and the variation of the total Donnan electric potential is as follows:

(45) $$\Delta \phi \;=\;\frac{R\;T}{F}\;{\left(\frac{c_{0L}}{X}\right)}^{3}$$

## 4. Conclusions

A simplified theoretical analysis of the thermodynamic equilibrium governing the ionic transport through IEMs under RED conditions has been presented and particularly analysed for both symmetric (1:1) and asymmetric (2:1) electrolytes. By examining the equilibrium conditions at the membrane–solution interfaces and the associated Donnan ion partitioning, approximate analytical expressions for the counter-ion concentration gradients inside the membrane have been successfully derived.

This study highlights that the ion partitioning coefficient strongly depends on the bathing ionic concentrations, on the membrane fixed-charge concentration, and on the asymmetry of the electrolyte. Therefore, the common assumption of the total co-ion exclusion is not meaningful in permeable membranes under RED conditions, especially when the bathing solutions exhibit large salinity differences.

The derived approximate expressions correspond to small deviations of the counter-ion concentration with respect to the ideally selective behaviour, and they provide useful theoretical tools to estimate ionic concentrations and Donnan potential differences within IEMs with realistic values of the fixed-charge concentration, without relying on complex exact or numerical solutions. The proposed formulations are the result of practical interest for the development of simplified and easy-to-implement models of electro-membrane processes. This approach will allow us to get a clearer physical interpretation of how membrane fixed-charge concentration and electrolyte asymmetry influence the energy conversion efficiency in RED systems.

## Figures and Tables

**Table 1:** Comparisons of exact and approximate values, including relative errors, for ionic concentrations, variations of the total Donnan potential, and ionic partitioning coefficients in a 1:1 system with *c*_0*L*_ = 0.5 M and *c*_0*R*_ = 0.02 M, for the membrane fixed-charge concentrations *X* = 1, 3, and 5 M.

*X* (M)		*c*_1_*_L_* (M)	*c*_1_*_R_* (M)	∆*c*_1_ (M)	∆*ϕ* (*RT*/*F*)	*k_L_*	*k_R_*
*X*		$X\;+\;\frac{c_{0L}^{2}}{X}$	*X*	$\frac{c_{0L}^{2}}{X}$	${\left(\frac{c_{0L}}{X}\right)}^{2}$	$\frac{X}{c_{0L}}\;+\;\frac{c_{0L}}{X}$	$\frac{X}{c_{0R}}$
1	Ex.	1.207	1.000	0.207	0.180	2.414	50.02
	Ap.	1.250	1.000	0.250	0.250	2.500	50.00
	% Err.	3.6	<0.1	20.9	33.1	3.6	<0.1
3	Ex.	3.081	3.000	0.081	0.027	6.162	150.01
	Ap.	3.083	3.000	0.083	0.028	6.167	150.00
	% Err.	<0.1	<0.1	2.9	4.3	<0.1	<0.1
5	Ex.	5.050	5.000	0.049	0.010	10.10	250.00
	Ap.	5.050	5.000	0.050	0.010	10.10	250.00
	% Err.	<0.1	<0.1	1.2	1.6	<0.1	<0.1

**Table 2:** Comparisons of exact and approximate values, including relative errors, of the ionic concentrations for the 2:1 salt, the variation of the total Donnan potential, and the ionic partitioning coefficients in a system with *c*_0*L*_ = 0.5 M and *c*_0*R*_ = 0.02 M for the fixed-charge *X* = 1, 3, and 5 M.

*X* (M)		*c*_1_*_L_* (M)	*c*_1_*_R_* (M)	∆*c*_1_ (M)	∆*ϕ* (*RT*/*F*)	*k_L_*	*k_R_*
*X*		$\frac{X}{2}+\sqrt{\frac{c_{0L}^{3}}{4X}}$	$\frac{X}{2}$	$\sqrt{\frac{c_{0L}^{3}}{4\;X}}$	$\frac{1}{2}\;{\left(\frac{c_{0L}}{X}\right)}^{3/2}$	$\sqrt{\frac{X}{c_{0L}}+\sqrt{\frac{c_{0L}}{X}}}$	$\sqrt{\frac{X}{c_{0R}}}$
1	Ex.	0.655	0.500	0.153	0.133	1.618	7.081
	Ap.	0.677	0.500	0.177	0.177	1.645	7.071
	% Err.	3.4	<0.1	15.5	31.7	1.7	<0.1
3	Ex.	1.599	1.501	0.098	0.032	2.529	12.25
	Ap.	1.602	1.500	0.102	0.034	2.531	12.25
	% Err.	<0.1	<0.1	4.1	7.5	<0.1	<0.1
5	Ex.	2.578	2.501	0.078	0.015	3.211	15.81
	Ap.	2.579	2.500	0.079	0.016	3.219	15.81
	% Err.	<0.1	<0.1	1.8	3.4	0.2	<0.1

## Data Availability

The raw data supporting the conclusions of this article will be made available by the author on request.

## Appendix A. Generalised Expressions for a z_1_:z_2_ Electrolyte (z_1_c_1K_ = −z_2_c_2K_ = c_0K_)

Total co-ion exclusion hypothesis	
$k_{K}^{z_{1}}\;-\;\frac{X}{c_{0K}}\;=\;0$	$c_{1K}\;=\;c_{10}\;k_{K}^{z_{1}}\;=\;\frac{X}{z_{1}}$
Ion partitioning equations	
$k_{K}^{z_{1}\;-\;z_{2}}\;-\;\frac{X}{c_{0K}}\;k_{K}^{-\;z_{2}}\;-\;1\;=\;0$	$c_{1K}^{\frac{z_{1}\;-\;z_{2}}{z_{1}}}\;-\;\frac{X}{z_{1}}\;c_{1K}^{-\;\frac{z_{2}}{z_{1}}}\;=\;{\left(\frac{c_{0K}}{z_{1}}\right)}^{\frac{z_{1}\;-\;z_{2}}{z_{1}}}$
Expansions	
$c_{1K}^{\frac{z_{1}\;-\;z_{2}}{z_{1}}}=\;{\left(\frac{X}{z_{1}}\;+\;y_{K}\right)}^{\frac{z_{1}\;-\;z_{2}}{z_{1}}}=\;{\left(\frac{X}{z_{1}}\right)}^{\frac{z_{1}\;-\;z_{2}}{z_{1}}}\;+\;\frac{z_{1}\;-\;z_{2}}{z_{1}}\;{\left(\frac{X}{z_{1}}\right)}^{-\;\frac{z_{2}}{z_{1}}}\;y_{K}$	
$c_{1K}^{-\;\frac{z_{2}}{z_{1}}}=\;{\left(\frac{X}{z_{1}}\;+\;y_{K}\right)}^{-\;\frac{z_{2}}{z_{1}}}=\;{\left(\frac{X}{z_{1}}\right)}^{-\;\frac{z_{2}}{z_{1}}}\;-\;\frac{z_{2}}{z_{1}}\;{\left(\frac{X}{z_{1}}\right)}^{-\;\frac{z_{2}}{z_{1}}\;-\;1}\;y_{K}$	
Counter-ion concentrations	
$c_{1L}\;=\;\frac{X}{z_{1}}\;+\;y_{L}\;=\;\frac{X}{z_{1}}\;+\;\frac{X}{z_{1}}\;{\left(\frac{c_{0L}}{X}\right)}^{\frac{z_{1}\;-\;z_{2}}{z_{1}}}$	$c_{1R}\;=\;\frac{X}{z_{1}}$
Counter-ion concentration gradient	
$\Delta c_{1}\;=\;c_{1L}\;-\;c_{1R}\;=\;\frac{X}{z_{1}}\;{\left(\frac{c_{0L}}{X}\right)}^{\frac{z_{1}\;-\;z_{2}}{z_{1}}}$	
Variation in total Donnan electric potential	
$\Delta \phi \;=\;\frac{R\;T}{z_{1}\;F}\;{\left(\frac{c_{0L}}{X}\right)}^{\frac{z_{1}\;-\;z_{2}}{z_{1}}}$	
Ionic partitioning coefficient	
$k_{L}\;=\;{\left[\frac{X}{c_{0L}}\;+\;{\left(\frac{c_{0L}}{X}\right)}^{-\;\frac{z_{2}}{z_{1}}}\right]}^{1/z_{1}}$	$k_{R}\;=\;\frac{X}{c_{0L}}$
